# The Reality of Healthcare Professionals in Leadership Positions at the Onset of the COVID-19 Pandemic

**DOI:** 10.3390/ijerph21091154

**Published:** 2024-08-30

**Authors:** Nancy Shehadeh, Georgina Silva-Suarez, Emily Ptaszek, Farah Roman Velez

**Affiliations:** 1 Department of Health Administration, College of Business, Florida Atlantic University, Boca Raton, FL 33431, USA; 2 Department of Sociobehavioral and Administrative Pharmacy, Nova Southeastern University, San Juan, PR 00926, USA; gsilvasuar@nova.edu; 3 Community Health Partnership, Colorado Springs, CO 80903, USA; 4 Barry and Judy Silverman College of Pharmacy, Nova Southeastern University, San Juan, PR 00926, USA; fr373@mynsu.nova.edu

**Keywords:** COVID-19, healthcare professionals, leaders, leadership, qualitative, mental health, crisis management, healthcare professional burnout

## Abstract

While pandemics have long been a topic of discussion in public health, COVID-19 placed healthcare leaders in a completely new and challenging situation. This qualitative study sought to understand the personal experiences of healthcare professionals in leadership roles at the beginning of the COVID-19 pandemic. Sixteen in-depth interviews were conducted and recorded via Zoom. Most participants were men (*n* = 8, 57.1%) and had a doctorate or master’s degree (*n* = 8, 57.1%). The themes of mental health, dynamic infrastructure, and transformative experience emerged from our participants’ narratives. Most respondents reported heightened stress during that time and shared the institutional and personal mechanisms they used to deal with the situation. They were proud of their profession and their work. They discussed the “dynamic infrastructure” they experienced at the time that helped them lead. Feeling overworked was a common experience for them. Most considered leading during COVID-19 a “transformative experience” that taught them valuable lessons. They also witnessed acts of heroism as their colleagues continued to work during difficult times, even though some succumbed to COVID-19. Despite all the challenges and uncertainties healthcare professionals in leadership positions faced at the onset of COVID-19, their resilience, dedication, and commitment to their profession prevailed. In conclusion, the firsthand experiences recounted by healthcare leaders in this study shed light on the multifaceted nature of leadership during a global health crisis. Their unwavering resilience, dedication, and commitment stand as a testament to the fortitude required in such demanding circumstances. As the COVID-19 pandemic continues to evolve, the insights gleaned from this research bear significant implications for informing future strategies and support systems aimed at bolstering healthcare leadership worldwide.

## 1. Introduction

As the SARS-CoV-2 virus spread rapidly across the globe, the COVID-19 pandemic plunged the healthcare sector into a state of uncertainty and fear [1]. Healthcare professionals in strategic leadership roles, along with their staff, faced immense pressure as the world relied on them to save lives and ensure public safety despite the unknown magnitude of the threat posed by the virus. It was similar to previous coronaviruses yet novel in many ways. Even though pandemics have often been discussed in the public health arena, COVID-19 put healthcare leaders in a position they had not experienced before [2]. No one was truly prepared for what was to come.

Strategic leadership is the type of leadership that happens at the highest level of an institution. Strategic leadership is defined as “the functions performed by individuals at the top levels of an organization that are intended to have strategic consequences for the organization” [3]. Positions at this higher level include chief executive officer (CEO), chief financial officer (CFO), chief operating officer [COO], chief medical officer, and executive director, among others. An unprecedented event challenged individuals in these roles at the onset of the COVID-19 pandemic. 

Healthcare leaders were put in a position where they needed to lead their frontline healthcare staff and their healthcare organizations without any clear protocols on the most effective way to lead in such a trying time [4]. They found themselves working longer hours than they had ever imagined and needing to connect with their frontline healthcare workers in a way that they had never had to do before. Healthcare staff were exposed to significant stressors, and many began experiencing mental health problems due to the challenges of the societal shutdown along with the unexplained high mortality linked to COVID-19 illness (especially during 2020, 2021, and early 2022) [5]. As Greenberg and Tracy [6] noted, to effectively support their healthcare staff, leaders need to follow a preventative medicine model, being candid about the information being shared and emphasizing the importance of the healthcare professionals as well as the organization’s commitment to them. 

When analyzing leadership strategies amidst the pandemic, significant fluctuations in decision-making become apparent. Sweden, for instance, bore substantial consequences, particularly concerning their elderly population, as they opted against implementing a lockdown. Conversely, New Zealand adopted a meticulous and transparent approach, exemplified by its stepwise escalation and de-escalation plan. This strategy not only assuaged workforce anxieties but also demonstrated effective crisis management. Furthermore, amidst escalating tensions, leaders were tasked with addressing issues of racism, discrimination, and aggression, compounded by the challenges of employees grappling with depression and anxiety [7,8].

Effective communication is essential for healthcare leaders during any crisis, including the COVID-19 pandemic [9,10]. Leaders must connect with all levels of the organization during emergencies to ensure everyone understands the necessary actions and their implications. The COVID-19 crisis has highlighted the need for strong interpersonal relationships and open communication to foster trust within teams. A study on the leadership team at West Virginia University Hospitals and Health System underscored the importance of effective communication in conveying the urgency of the pandemic. It also highlighted the need to empower staff to act on the vision of and collaborate effectively as key contributors to the COVID-19 plan [11].

In times of workplace uncertainty, strategic leadership must follow a clear plan supported by an agile mindset. According to Arif Nazir [12], healthcare leaders have learned five pivotal lessons from the pandemic covering a range of areas, including communication strategies and emergent innovations. The first lesson underscores the moral imperative inherent in healthcare decision-making. Lesson two underscores the necessity for leaders and healthcare professionals to champion discussions regarding geriatric education and the philosophy of care, while lessons three and four stress the importance of effective communication and fostering trust among workers. Lastly, the pandemic has spurred a wave of innovation to enhance telehealth and shared-savings programs [12].

The experiences of strategic leadership personnel during COVID-19 are unique and invaluable. Their insights, fears, challenges, and triumphs provide crucial information on the factors that helped high-level leaders in health organizations navigate the pandemic. This paper explored healthcare leaders’ lived experiences at the onset of COVID-19.

## 2. Methods

The authors conducted a qualitative study using interpretative phenomenological analysis (IPA) [13] to explore the life experiences of healthcare professionals in leadership positions at the onset of the COVID-19 pandemic through in-depth interviews. Three authors developed a question guide (N.S., G.S.S., E.P.). N.S. is a Middle Eastern female public health researcher who has worked with marginalized populations, focusing on HIV risk and intravenous drug use, as well as health outcomes. G.S.S. is a Hispanic female researcher with an educational background in public health who has worked with vulnerable populations and public health emergencies, such as natural disasters. She has conducted various qualitative research projects and has had formal and informal training in conducting qualitative interviews and focus groups. E.P. is a Caucasian female with an educational background in psychology and business who has worked with vulnerable populations both clinically and in leadership positions. She has also led a Federally Qualified Health Center during the COVID-19 pandemic. IPA in this study involved discussing the list of questions with potential participants and co-researchers. After discussing and agreeing on the interview guide with the research team, the question guide was piloted with a potential participant. The pilot interview was conducted to ensure that we collected the necessary information to answer our main research questions. Following the pilot, the participants approved the order and sequence, and no changes were made. The Nova Southeastern University Institutional Review Board approved the study. 

A purposeful sampling snowball technique was used to recruit our study participants. The inclusion criterion for potential participants was that they had a healthcare leadership position at the onset of the COVID-19 pandemic. Potential participants were identified and invited to participate in the study via email. Healthcare leaders were also asked to refer other leaders, and some were contacted through LinkedIn based on their positions as CEO, COO, or CMO. If they agreed to participate, another email with the informed consent form was sent, and an interview date was set up. Any doubts or concerns based on the information provided about the study were discussed with the first author (N.S.). Study participants also completed a demographic questionnaire on the interview day. More than 20 potential participants were reached out to and invited to take part in the study. Eighteen agreed to participate. Two of those did not show up at the time of the interview. Our final sample was 16. Recruitment ended after interviewing 16 participants as we had gathered all the necessary information for our study objective and obtained no new insights, themes, or patterns from further data collection, clearly indicating that saturation was achieved. Participants were located in Florida, New York, California, and Pennsylvania. Most held clinical degrees and worked at healthcare organizations with over 300 employees. 

The interviews were conducted between November 2021 and March 2022. During this period, the United States experienced a significant increase in daily COVID-19 cases, exceeding 800,000 daily cases [14]. Most infections were attributed to the Omicron variant, known for its higher transmissibility compared to other COVID-19 variants. In December 2021, California and New York, the first regions in the U.S. where Omicron was detected, recorded a combined COVID-19 death toll that surpassed 800,000. By January 2022, New York state had reported its highest number of new COVID-19 cases in a single day since the pandemic began, with 114,082 confirmed cases. At that time, the U.S. saw nearly one million new COVID-19 infections in a single day, the highest daily total worldwide. Additionally, the number of hospitalized COVID-19 patients increased by nearly 50% in just one week [15].

Interviews were conducted via Zoom, lasting approximately thirty to sixty minutes. They were recorded and transcribed verbatim by a professional. Quirkos, a qualitative data manager and analysis software program, was used to facilitate the analysis. Three members of the research team coded the interviews. After all the interviews were coded, the codes were revised by N.S. and G.S.S. When discrepancies were found, both authors discussed them and reached an agreement, always considering the voices of the study participants.

**Figure 1:**
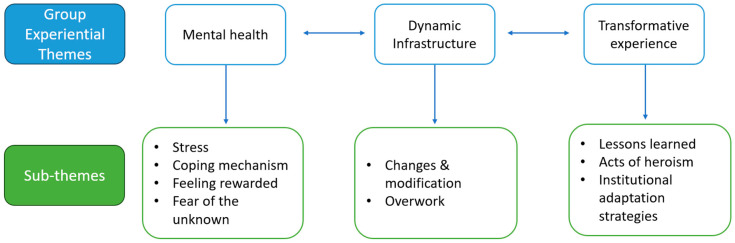
Graphic representation of the data analysis process.

Transcripts were cleaned by removing any comments that may have compromised confidentiality and later were examined individually following the steps proposed by Smith [13]. No themes were identified a priori. Part of the research team read the transcripts several times to ensure that they captured the participants’ thoughts accurately. Initial codes emerged from this process. *Experiential statements* were identified as the ones that best represented our participants’ lived experiences as healthcare professionals in leadership positions at the onset of the COVID-19 pandemic. Later, ‘*Personal Experiential Themes*’ were developed using the experiential statement, and this was performed through the analysis of a single case (by participants). ‘*Group Experiential Themes*’ were developed by looking at patterns, relationships, convergence, and divergence among cases. Three ‘Group Experiential Themes’ were identified: mental health, dynamic infrastructure, and transformative experiences (see Figure 1). These Group Experiential Themes were used to structure our findings. Direct quotes from our participants were added to illustrate the findings. Fictitious names were given to protect their identity. 

## 3. Findings

### 3.1. Demographic Characteristics of the Study Population

Most of our study participants were male (*n* = 8, 57.1%) and had a doctoral degree (*n* = 8, 57.1%). Their areas of expertise were diverse and included health administration, public health, social work, and medicine, among others. Half of our study participants had 11 to 20 years of work experience, 35.7% had 21 to 30 years of experience, and 14.3% had over 30 years of work experience (see Table 1). Although we interviewed 16 participants, only 14 completed the demographic questionnaire. The remaining two participants did not complete the questionnaire for unknown reasons.

### 3.2. Mental Health

#### 3.2.1. Stress

Stress emerged as one of the primary feelings that our participants experienced due to the uncertainty and lack of information about the novel SARS-CoV-2 virus and its effects on the population. Participants repeatedly commented about the high level of stress they endured every day and the methods they used to deal with day-to-day situations, including leading a team and ensuring the safety of every team member.

*Peter, Chief Operating Officer, stated, “The biggest concern was to make sure that our offices stayed open and our staff stayed safe. You know, the—that was our—I mean, our primary concern was, how do we operate? Because if we close our doors, folks don’t have anywhere to go”.*

The challenges they had to overcome included the lack of sufficient personal protective equipment for personnel. *Peter, also noted, “In the field, especially in the hospital-centered organization, a lot of times if the PPE was not available, services for patients had to continue”.*

In addition to work challenges, the participants were concerned about having to isolate themselves from their families to protect them from potential exposure. Study participants mentioned that the amount of work they had and the constant worry about the safety of all the people around them negatively affected their personal life and work–life balance.

*Peter, Chief Operating Officer, added, “So, during the pandemic, I mean, the height of it, there was no such thing as work-life balance. You know, there was being at work and dealing with the situations. And then, going home for a little bit and doing my best not to think about it, which was quite literally an impossibility”.*

#### 3.2.2. Coping Mechanisms

The study participants discussed how they managed all the daily stressors they experienced during the onset of the pandemic. Some used exercise as a coping mechanism, others said that being with their loved ones gave them peace, and still others went to the beach to experience the peace and calmness of the ocean. *Betty, Director of Pharmacy, shared, “Exercise. You know, thanks to my military background. That’s kind of always been a part of my life…”*

For Peter, running was the coping mechanism he used, and he described it as “therapeutic”. He shared,

*“…I had picked up running before the pandemic hit. You know, knowing I couldn’t gather with anyone, I ran. It was my moment … Therapeutic is the right word, but sort of, it was distressing…”*

Leila, a Chief Operating Officer, preferred to stay at home with her family while she was not working. She shared with us,

*“I was just kind of in my house with my son and my husband and just kind of in a cocoon until I had to wake up the next day and go to work, right? And so, like being home, was a sense of peace and calmness for me. And it gave me so much bonding time…”*

Cindy, Senior Vice President and Chief Nursing Executive, found peace at the beach. She narrated,

*“So yeah, so for me, the beach is my thing. Not necessarily going into the water, just to be, you know, sitting back listening to the waves, watching the birds come down and get the fish out of the water…”*

#### 3.2.3. Feeling Rewarded

Despite all the challenges, our participants still felt proud, blessed, and satisfied with the career they had chosen. They said that they would not be in any other position. Some of them indicated that it also reassured them of their purpose and their will to continue in this line of work. One of our participants shared,

*“This [experience] has solidified more my decision to be in healthcare. I mean, it literally is the most, I think, rewarding field that a person can work in. Even though it’s one of, I think, the most difficult areas to work in right now, there’s not a day that goes by that I don’t know that I’m making a difference in my career” (Betty, Director of Pharmacy).*

As healthcare leaders, participants felt compelled to act and help in any way possible. As Alice, Chief Operating Officer, shared,

*“I felt that I had a greater responsibility to the people at that point. And—I don’t mean in this kind of cliche way. I mean, actually, I couldn’t sit, stay in my office. We had to go to the COVID floor. So those staff members knew that we not only appreciated them, but we also knew the fear they were experiencing, and so we were with them”*.

#### 3.2.4. Fear of the Unknown

Some study participants expressed a “fear of the unknown”, and the lack of accurate information affected how our participants led their organizations. Additionally, the pressure and rapid institutional demands from administrators influenced the changes implemented in our participants’ organizations. The quote below illustrates this struggle, noted by more than one participant in our sample.

*Adam, Director of Pulmonary and Critical Care Medicine, stated, “So, there was fear. There was, a lack of knowledge. There was structural demand that required us to change the care delivery systems. And, you know, we had to adapt to all of that in a comprehensive and well-thought plan”.*

Several participants also noted influential moments that created fear and hesitancy among the staff due to the level of uncertainty and fear of the unknown effects of the SARS-CoV-2 virus.

*Adam, Director of Pulmonary and Critical Care Medicine, shared, “I think the biggest, most impactful moment was when a written statement went out to all the clinicians saying, you can be virtual when you can be virtual, but there’s no getting around it that you’re going to have to be at the bedside. And we’re going to do everything that we can to protect you. And we’re going to consider all the factors before we send you. But you are going to be at the bedside. Nurses across the country are making contact with their patients. And we can’t stay virtual in this business”.*

Many healthcare workers had to make difficult decisions. Some quit their jobs because they did not want to risk putting the lives of their families in danger. This also put additional stress, fear, and strain on other workers because of the increased amount of work and limited personnel numbers, which led to fatigue, exhaustion, and burnout.

*Leila, Chief Operating Officer, explained, “…We had some people that chose to leave, got really scared, especially when we didn’t have any PPE. I mean, what can you say to people who were walking off the job, saying, “I can’t do this anymore” or “I cannot put my family at risk? I have people at home who are vulnerable adults”. Staff who had other illnesses created an additional vulnerability. And, you know, we just [told them], “the door’s open for you to come back. We understand, and they had to leave us…”*

### 3.3. Dynamic Infrastructure

All study participants noted that the COVID-19 pandemic changed their daily behaviors and lifestyles, organizational operations and protocols, roles at work, and, in essence, every part of their daily lives. The COVID-19 pandemic required them to dig deep into their emergency preparation/coping skills toolkit and utilize such skills to lead their organization during these uncertain times without any guidance or real preparation on how to handle the day-to-day challenges that arise during a pandemic in a healthcare organization.

#### 3.3.1. Changes and Modifications

One of the major concerns most participants noted was the constantly changing environment during the early part of the pandemic, coupled with fear of the unknown. The participants reported that, at the beginning of the pandemic, healthcare organizations kept a close eye on the new cases that were being reported. They were well aware that it was only a matter of time until new cases began popping up in their hospitals. This motivated healthcare administrators to begin the preparation phase, which involved changing the role of healthcare administrators, directors, and other staff depending on what was needed and considering the emergency preparedness skills they could bring to the table.

*Jamal, Chief Medical Officer, shared, “So I gathered my team at the hospital. One of my guys, one of my associate medical directors, is big into emergency management. So, I said, you know, I need you to develop a standard operating plan because it’s going to change every day”.*

Notably, many of the clinical staff resigned shortly after the pandemic began. Healthcare leaders needed to be strategic in how they led their organizations. One crucial strategy, according to several healthcare administrators, was to focus on being transparent and genuine to win the trust of their staff and patients. This required them to admit that everyone is scared (including themselves) and that everything is being carried out (to the best of their ability) to protect the clinical staff as they treat COVID-19-positive patients as well as all patients residing in the hospital. One of our study participants shared, 

*“But I was conscious of the fear that a lot of people have. Right? And so that’s something we really focused on. Like, yeah, it’s, you know, we admitted we were all scared. And I think that that was a big part of it. Initially, it is to say it’s okay to be scared. I’m scared. You’re scared. We’re all scared. Like, there’s nothing we can do…Right” (Cindy, Senior Vice President and Chief Nursing Executive).*

Adam, the Director of Pulmonary and Critical Care Medicine, who played a pivotal role in educating the healthcare professionals at his healthcare organization about the COVID-19 illness, shared a similar sentiment:

*“And also, again, there was the challenge of getting people on board. I’m not going to lie to you. There was a lot of fear, you know, whether us because we’ve not known much, and we were concerned about them. And I remember in January, you know, we start hearing this report about the emergency doctor who died, the anesthesiologist who died, the nurses who died. So, there was also fear”.*

Healthcare professionals learned something new every day about how the virus was being transmitted, who was most susceptible to the virus, and what were the most effective ways to treat it. Our interviewees agreed that everyone was scared, regardless of their position. 

Most participants said that a command center focused on COVID-19 planning was created at their facility, with leaders pulled from different departments.

*Sam, Director of Operations, stated, “When we received directions from the state, declaring the state of emergency and the WHO announcing the pandemic, when that occurs, that initiates the hospital Incident Command Center. So, when the CEO announced that the Incident Command Center was going to be launched, all executives had to follow a specific process. Per the incident command, we assigned the responsibilities. So, the COO, the chief operating officer, was responsible as the [leader of] incident command, and then there were responsibilities given to each executive”.*

Most leaders noted that, for the first time, they were exploring and implementing strategies to keep their staff and healthcare professionals motivated and appreciated for all they do. Different participants noted that they tried to connect with their staff and the medical staff to see how they were feeling and chat about how their day was going. Non-medical staff were asked to fulfill different roles due to additional departments being created, such as COVID-19 testing centers, and the leaders had to make sure they operated efficiently. Then, later on during the pandemic, vaccination centers (some in a drive-thru format) were created and operated.

#### 3.3.2. Overworked

Some of our study participants noted that individuals in leadership positions felt overwhelmed and overworked. The response phase of the COVID-19 pandemic was intense, and most did not feel they had a break or a good balance between their professional and personal lives. As one study participant shared, some decided to change professions after recognizing the toll of the pandemic on healthcare workers’ mental health:

*“And that’s where I think we see a lot of the burnout and folks, you know, changing profession or leaving the profession because, you know, we didn’t, we didn’t perhaps recognize early enough the toll it was going to have on leaders’ mental health in the healthcare profession” (Alice, Chief Operating Officer).*

Others noted it was difficult to take a break or even think about themselves as their leadership position required them to prioritize their work responsibilities over their well-being due to the public health emergency.

*Jamal, Chief Medical Officer, shared, “I think it’s very tough for leaders to monitor themselves during events like this, and again, there’s that sense, this is a crisis. I need to lead. You know, I don’t get to take a break. For many of us, including myself, unfortunately, that’s the feeling like, well, this is too important”.*

A few participants noted that they needed “constant check-ins” with their healthcare professional team to make sure they were not burned out, and, if they were, they did their best to try to address the symptoms. This is what one of our study participants shared:

*“So, it’s not easy when you are doing lengthy ICU shifts, and then you get through this wave, you survive it, you celebrate it, you throw a party for the fellow and the rest thanking them for their effort. And then you get another wave in two months. So, that led to, you know, burnout, fatigue, sleep deprivation. So, I was faced with the responsibility to screen for those things that in the past, you know, probably, we would talk about it but not in a systematic fashion. Now, I feel that I have to have this conversation with my trainee, “Hey, how are you doing? How are you feeling? Let me take—let me see the hours that you logged in. You logged in extra, you know, let’s say 70 to 80 h per week compared, let’s say, in the past few months, you were logging in 40 to 50 h per week. You are working hard, and I can see that. But how do you feel about that? Do you need a couple of days of vacation? How can I destress you?” (Adam, Director of the Pulmonary and Critical Care Medicine Fellowship Program).*

### 3.4. Transformative Experience

#### 3.4.1. Lessons Learned

Most study participants agreed that, despite all the hardship, the COVID-19 pandemic was transformative and a learning experience in many ways. They described how, at the beginning of the pandemic, facing the unknown and having very little information regarding COVID-19, they continued to provide services to their community. In addition, they highlighted the importance of teamwork and communication. As Sam shared,

*“You know, because what I learned is that I got a lot of support. And although I’m the operations guy, and I’m supposed to have all the answers to everything, I didn’t have all the answers. But, you know, when I reached out to my leadership group, and I reached out to my team, I got some great feedback where I was able to make good decisions. So, you must engage your team, engage your leadership, and engage as many supporters as you can to help you make decisions, especially in unprecedented times like that where it’s very challenging and steadfast” (Director of Operations).*

#### 3.4.2. Act of Heroism

Some study participants shared how difficult it was to lose part of their staff due to COVID-19 and how they felt responsible for their staff’s deaths, praising these workers as heroic. Lisa, an Executive Director, shared,

*“I guess one thing I’ll never forget is, when we lost [Lucy, fictitious name]. She was a hospice aide who worked at our unit. …everything happened so quickly. She came to work on a Saturday. And by the following Wednesday, she was gone. So, she tested positive for COVID-19 on that Monday. So, what I take away and what I remember is that the good stuff, right, it’s the stuff that created all the turmoil, and it was so emotional, and I felt responsible, and, you know, that was hard”*. 

Lisa continued by discussing how they coped with the harrowing experiences of losing a co-worker.

*“But everybody came together. We honored Lucy. We did a virtual meeting. We had a picture up. The music therapist sang songs, played the guitar for us, we did mindfulness, we invited her family, we donated to her family, we did everything that we could think of to really make her important and to honor her as a healthcare hero, not just to say that, you know, but to really put that, what she did, what she did for us, you know, and how do you honor that then? That’s what I remember. And thank God we only lost one employee. Some companies lost multiple employees. And that’s a lot to deal with”*. 

#### 3.4.3. Institutional Adaptation Strategies

Healthcare professionals in leadership positions need to deal with a lot in times of uncertainty. Sam, one of our study participants, described “Code Lavender”, which his institution adopted as a special mechanism to assist staff during difficult times.

*“Code Lavender is a team of people that get called to a unit or to an area when employees are stressed or when there is a challenging time for them, whether it be a death in the family or the death of the patient that was, you know, something that occurred that had a negative outcome for them. This group consisted of a physician, a mental health counselor, as well as [others] who would meet with the team and talk to them and provide resources to help them”*. 

One participant highlighted how their leadership style was transformed and expanded to provide additional support to their employees. Some employees needed that reassurance from their leadership. 

*“So, I felt like, in a certain instance, I’m becoming a little bit of a caregiver. Not that you aren’t that in leadership, but more of a caregiver, being much more involved in the soothing, and supporting, bolstering, pumping up of staff”*. 

## 4. Discussion

The COVID-19 pandemic profoundly affected healthcare professionals in strategic leadership positions. This study explored the lived experiences of various healthcare leaders during the onset and ongoing evolution of the pandemic. Through in-depth interviews and interpretative phenomenological analysis, three experiential themes emerged: stress and mental health response, dynamic infrastructure, and transformative experiences. 

Notably, many respondents reported experiencing both a sense of meaning and purpose alongside overwhelming stress. A significant source of stress for most participants was the fear of contracting COVID-19 and transmitting it to their family members. This concern aligns with findings from a study by Alvarez [16], which examined how organizational healthcare leaders supported frontline workers. This brings up important questions regarding coping, post-traumatic stress vs. post-traumatic growth, and longevity post-pandemic among individuals in healthcare leadership roles. Those who felt more in touch with the meaning and purpose of their roles, despite the stress, may be more likely to sustain longer-term healthcare leadership careers. This relationship, as well as the relationship between leadership, career longevity, and the ability to emotionally process one’s experiences, needs to be explored in future studies.

Consistent with other studies [17,18], most of our study participants felt rewarded for working in the healthcare field at the onset of the COVID-19 pandemic. Their sense of moral and social responsibility towards the profession and their patients motivated our study participants to continue working tirelessly during difficult times. In another study, conducted in Pakistan [19], healthcare workers also stated that their motivation and professional attitudes extended beyond their job responsibilities to serving humanity. In a study conducted in Italy among healthcare professionals [17], the researchers found that having an intrinsic motivation towards their profession, social and moral responsibility, good relationships with their colleagues, and supportive management were protective factors during those difficult times.

One participant highlighted the need to “expand and transform” their leadership to provide additional support to their employees, noting that this period was particularly stressful, especially for younger healthcare professionals. Survey studies conducted in the U.S. by the Census Bureau (2020) across three time points (weeks 1, 5, and 10) found that symptoms of depression and anxiety were more pronounced among younger adults [20,21,22]. Additionally, respondents with lower incomes reported higher rates of anxiety and depression. Another global cross-sectional study found that younger individuals experienced greater psychological distress compared to older adults during the pandemic [23]. Magnavita et al. (2021) conducted a study among anesthetist residents and specialists, finding higher stress levels among residents younger than the specialists [24]. 

They noted that, at the beginning of the pandemic, residents might have felt there was a lack of understanding of the principles, practices, and decision-making processes during that time. This insight aligns with our study participants’ remarks about the need to act more as “caregivers supporting and bolstering their staff”. Leaders in higher hierarchical positions with more experience had to manage their teams, which were simultaneously dealing with uncertainty, conflicting communication, and challenging work settings. 

One study participant emphasized healthcare leaders’ responsibility to ensure their staff’s mental well-being so they can provide quality patient care. This involves regularly asking staff “how they’re doing” and “if they need help” and monitoring their work hours to prevent burnout. Similarly, Stanz and Weber (2020) stressed the importance of promoting staff resilience by having open conversations about their well-being and making regular check-ins a priority for pharmacy leaders [25]. During challenging times, such as the COVID-19 pandemic, healthcare leaders must prioritize mitigating psychological risks for all their personnel, with a special focus on healthcare professionals.

It is also helpful for healthcare leaders to understand the specific coping mechanisms various leaders employed and whether those tools have worked post-crisis. One study reported strategies implemented by two organizations to help their staff cope with the COVID-19 pandemic and the difficult times they experienced as healthcare professionals. One organization implemented “mental health days”, free days negotiated with the supervisor, and the other organization implemented “respite time”, during which employees had four hours a week to do whatever they wanted [16]. Other studies highlighted the importance of supporting staff well-being [26]. Similarly, in our study, participants mentioned the “Lavender Code”, implemented to help staff or health professionals deal with difficult situations.

Additionally, it is also important to evaluate the current level of burnout among leaders and examine any connections between their coping mechanisms and effectiveness, both during COVID-19 and in relation to pre-pandemic burnout levels. In a more recent study, WittKieffer (2022) examined executive burnout among 233 respondents in C-level roles. The study used the Mayo Clinic definition of burnout, where it is clinically defined as “a state of physical, emotional, or mental exhaustion combined with doubts about one’s competence and the value of one’s work” [27]. Alarmingly, 74% of respondents reported feeling burned out in 2022, compared to 60% when polled in 2018. Likewise, 93% of respondents believed burnout was negatively affecting their organization when asked this question in 2022, compared to 79% in 2018 [27]. In terms of the profound effects of COVID-19 on the future of healthcare, it is critically important to understand the risk and protective factors leading to burnout. While most studies on burnout have assessed frontline clinical staff, this study was the first to examine executive burnout. The current study provides significant insights into how these leaders navigated rapid change and unending stress during a long-term, poorly understood, and unprecedented threat. 

The future of healthcare leadership depends on more than just improved training in finance, operations, and administration. Stress management, healthy coping mechanisms, transparency, and authenticity are additional crucial factors. Due to the prolonged nature of the pandemic, this study highlights the importance of ongoing mental health support for healthcare leaders who may be experiencing continuous psychological effects after the pandemic. It emphasizes the importance of structured mental health resources and training on stress management tailored for healthcare leaders. Along with the heightened level of mental health support, healthcare organizations should also develop flexible and adaptive leadership models and have flexible protocols that can be activated in times of crisis. These elements will benefit both leaders and healthcare systems. To identify the needs of future leaders, it is essential to understand the experiences of those who remained in healthcare leadership and those who left during and after COVID-19. Accordingly, leadership development programs should incorporate experiential learning as well as resilience-building to help future leaders handle high levels of stress. Policymakers and healthcare administrators may be able to use these findings to address the challenges that healthcare leaders face. This study also stresses the need to develop leadership frameworks that can be adapted to various healthcare systems on a global scale to ensure that future leaders can fulfill the core mission of healthcare: care for our communities.

## 5. Conclusions

The COVID-19 pandemic profoundly affected nearly every aspect of the healthcare system. Our study revealed that the pandemic significantly affected the mental health of healthcare professionals in strategic leadership positions who faced increased stress and uncertainty. They discussed their experiences as leaders in a constantly changing environment, the effects of these changes on their practice, and feelings of being overworked. Despite the stressful conditions, most participants reported a sense of reward and purpose due to their crucial roles during the onset of the pandemic.

The data gathered highlighted the multifaceted challenges that healthcare leaders faced during the COVID-19 pandemic, as well as their resilience and dedication to their roles. Exploring communication, decision-making, and team dynamics can offer valuable insights for understanding past healthcare crises and preparing for future ones.

## 6. Study Strengths and Limitations

One strength of this study was the diversity of professionals who participated in strategic leadership positions. Despite having only 16 participants, they came from various backgrounds, positions, areas of expertise, and geographical areas, enriching the information gathered. A limitation of the study was the challenge of scheduling interviews with busy leaders. While some participants agreed to participate, we often had limited interview time due to their responsibilities, a restriction not always present in other qualitative studies.

## Figures and Tables

**Table 1:** Demographic Characteristics of the Study Population.

Demographic Characteristics	* *n* = 14 (%)
Sex	
Male	8 (57.1%)
Female	6 (42.9%)
**Ethnicity**	
White, non-Hispanic	6 (42.9%)
Hispanic or Latino	3 (21.4.%)
Black or African American	2 (14.3%)
Middle Eastern/North African	2 (14.3%)
Asian	1 (7.1%)
**Highest degree completed**	
Doctorate degree	8 (57.1%)
Master’s degree	5 (35.7%)
Bachelor’s degree	1 (7.1%)
**Do you have a clinical degree?**	
Yes	10 (71.4%)
No	4 (28.6%)
**Have you ever worked in a setting with direct contact with patients?**	
Yes	13 (92.9%)
No	1 (7.1%)
**Current position**	
Chief Executive Officer (CEO)	4 (28.6%)
Chief Operating Officer (COO)	3 (21.4%)
Chief Medical Officer	2 (14.3%)
Chief Health Officer	1 (7.1%)
Chief Nursing Executive	1 (7.1%)
Executive Director	1 (7.1%)
Program Director	1 (7.1%)
Senior Manager/Inpatient Pharmacy	1 (7.1%)
**How long have you worked in your current position?**	
Less than one year	5 (35.7%)
1–3 years	5 (35.7%)
4–10 years	2 (14.3%)
More than 10 years	2 (14.3%)

* Only 14 out of the 16 participants completed the demographic questionnaire.

## Data Availability

Data is unavailable due to privacy and ethical restrictions.
